# Functional analysis of a novel *FBN1* deep intronic variant causing Marfan syndrome in a Chinese patient

**DOI:** 10.3389/fgene.2025.1564824

**Published:** 2025-03-19

**Authors:** Qingming Wang, Fang Zhang, Xinlong Zhou, Hui Li, Juan Zhao, Haiming Yuan

**Affiliations:** ^1^ Key Laboratory for Precision Diagnosis and Treatment of Severe Infectious Diseases in Children, Dongguan Maternal and Child Health Hospital, Dongguan, China; ^2^ Huadu District People's Hospital, Guangzhou, China

**Keywords:** deep intronic variant, FBN1, Marfan syndrome, minigene, RNA splicing

## Abstract

Marfan syndrome (MFS MIM#154700), due to pathogenic variants in the *FBN1* gene, is an autosomal dominant connective tissue disorder, typically involving the skeletal, cardiovascular and ocular systems. Currently, over 3000 MFS patients were reported, and approximately 1800 pathogenic variants in *FBN1* were identified. However, the molecular diagnosis still remains challenging for 8%–10% of patients with clinical features suggestive of MFS. In this study, we reported a 2-month-old Chinese female patient whose clinical features were compatible with the MFS. Whole-exome sequencing (WES) identified a novel *de novo* deep intronic variant, c.4943-8_4943-7insTATGTGATATTCAT TCAC in intron 40 of *FBN1* that was predicted to affect the RNA splicing. Minigene analysis showed that this variant causes skipping of exon 41, leading to the deletion of 41 amino acids (c.4943_5065del, p.Val1649_Asp1689del). It confirmed the pathogenic nature of the variant and established the genotype-phenotype relationship. Our study expands the mutation spectrum of *FBN1* and emphasizes the importance of deep intronic variant interpretation and the need for additional functional studies to verify the pathogenicity of these variants.

## Introduction

Marfan syndrome is a clinically recognized genetic disorder involving multiple congenital anomalies. Three cardinal manifestations are frequently seen in MFS patients and can be used as diagnostic clues, including skeletal anomalies (tall, thin stature, disproportionately long limbs and digits, anterior chest deformity and scoliosis), cardiovascular problems (mitral valve prolapse, mitral regurgitation, dilatation of the aortic root, and aortic regurgitation), and ocular anomalies (ectopia lentis and myopia). Aneurysm of the aorta and aortic dissection are the major life-threatening cause. MFS is caused by pathogenic variants in *FBN1* (encoding fibrillin-1) (MIM # 134797) (Pyeritz, 2000). To date, more than 3,000 patients with MFS have been reported in the literatures and 1800 pathogenic variants in *FBN1* have been identified (Collod-Béroud et al., 2003; Landrum et al., 2018; Arnaud et al., 2021); HGMD database. Pathogenic or likely pathogenic variants in coding exons and canonical splicing sites in *FBN1* could account for the majority of individuals with clinical features suggestive of MFS (Dietz et al., 2001). However, it was found that 8%–10% of individuals with suspected MFS remain genetically unexplained (Loeys et al., 2004; Baetens et al., 2011; Zeigler et al., 2021). It is partly attributed to some cryptic variants, such as noncanonical splicing variants, that may be missed during variant interpretation because they were generally considered to be non-deleterious effect on protein products. Furthermore, it is laborious to carry out functional studies in the lab on variants suspected of being a potential genetic cause of patients with MFS phenotypes.

In this study, we reported a 2-month-old female patient who displayed characteristics typical for MFS. Whole exome sequencing (WES) was performed for the patient and identified a novel *de novo* deep intronic variant (NM_000138.4: c.4943-8_4943-7insTATGTGATATTCATTCAC) in *FBN1*.

## Materials and methods

### Ethical compliance

This study was approved by the Ethics Committee of Dongguan Maternal and Child Health Hospital (DMCH 202307) and was performed in accordance with the Declaration of Helsinki. Written informed consent was obtained from the legal guardian for the release of any potentially identifiable image or data contained in this paper.

### Whole exome sequencing and sanger sequencing

Genomic DNA was extracted using nucleic acid extraction reagents according to the kit instructions. Whole exome sequencing (WES) was used to screen for causal variants in this patient. Sequencing was performed with an Illumina NovaSeq 6,000 (Illumina, San Diego,CA, United States). The bcl2fastq2 Conversion Software (v2.20) was applied for extracting Fastq files, and all reads were mapped to the human genome (GRCh37/hg19) by using BWA (v0.2.10) with default parameters. The Genome Analysis Toolkit (GATK; v.3.7) HaplotypeCaller was performed for identifying variants. The aligned reads were visualized by using the Integrated Genome Viewer (IGV). Common variants were filtered based on their frequencies in the databases of the Genome Aggregation Database (https://gnomad.broadinstitute.org/) and our internal database. The suspected variant was verified by Sanger sequencing. The pathogenicity of the sequence variants was interpreted according to ACMG/AMP guidelines (Richards et al., 2015).

### *In vitro* minigene assays

Wild type and mutant minigene plasmids were constructed for the *FBN1* variant (c.4943-8_4943-7insTATGTGATATTCATTCAC) using the exon trap vectors pcMINI. The sequence of exon 41 (123 bp), part of intron 40 (463 bp), and part of intron 41 (496 bp) were amplified from the proband’s or her mother’s genomic DNA, using the following primer pairs: forward 5‘-GGT​AGG​TAC​CGA​GTG​CAA​TGG​CAT​GAT​CTT-3’ and reverse 5‘-TGCAGAATTC TAC​CTA​TGC​TGC​TAC​AAG​AT-3’. The amplified products were inserted into the pcMINI vector. Then, plasmids were constructed and transfected into human embryonic kidney 293T (HEK 293T) and human breast cancer cells (MCF-7) respectively, in triplicates using Lipofectamine 2000 (Invitrogen, USA). Cell cultures were operated according to the literature previously published (Li et al., 2021). After 48 h of transfection of cells, the total RNA was extracted using TRIzol reagent (Cowin Biotech Co., Jiangsu, China). For RT-PCR, a pair of primers was designed to amplify the target sequence originated from the expressed minigenes: forward 5‘-CTA​GAG​AAC​CCA​CTG​CTT​AC-3’ and reverse 5’-TAG​AAG​GCA​CAG​TCG​AGG-3’. Finally, the PCR product was verified by Sanger sequencing and visualized with electrophoresis on a 1.2% agarose gel.

## Results

### Case presentation

**FIGURE 1:**
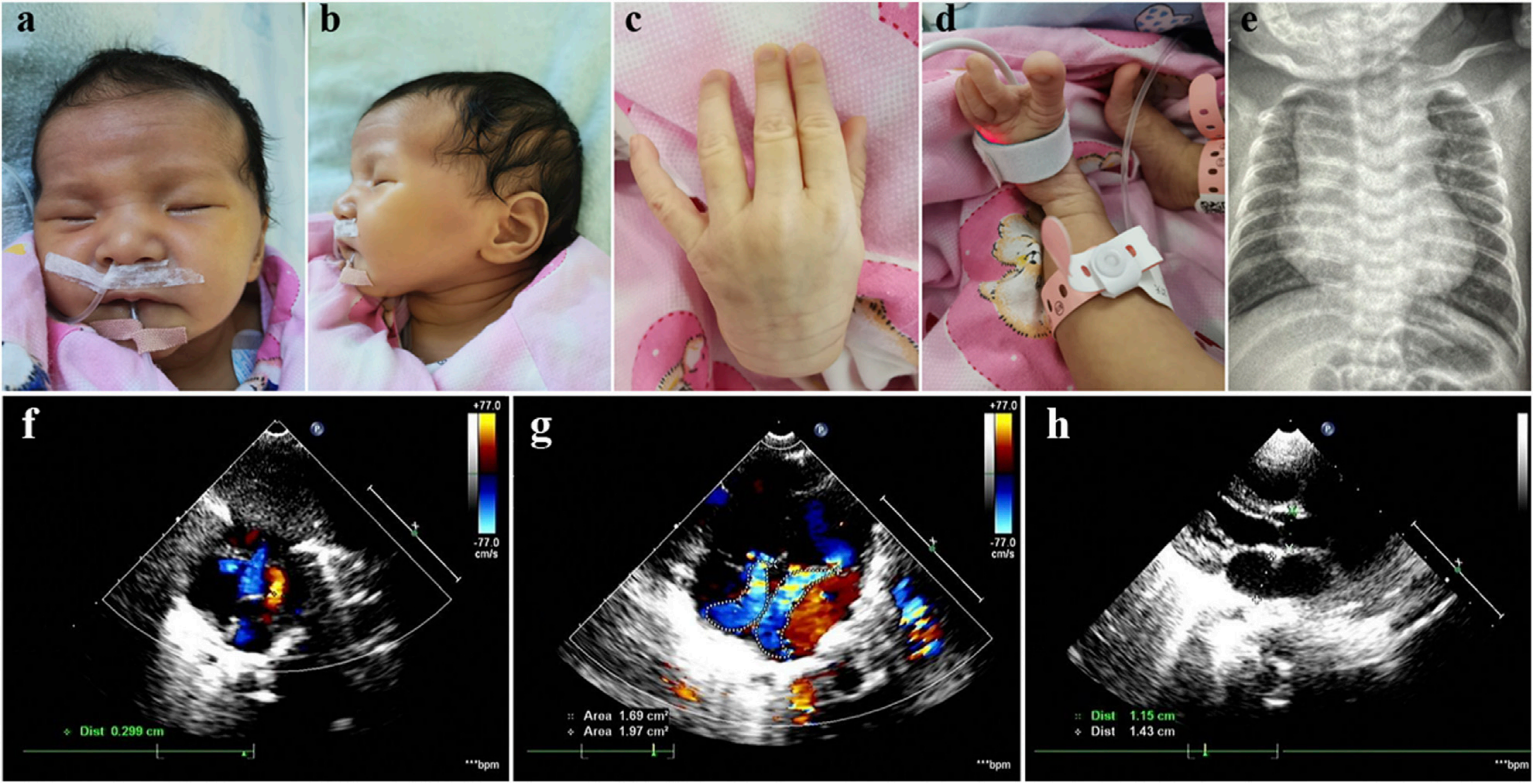
Photographs of our patient with MFS. Note bilateral temporal skull flattening, enophthalmos, retrognathia, arachnodactyly, pes planus, long, narrow feet, hammer toes and skin striae. **(A–D)** X-ray showed significant enlargement of the heart shadow with a cardiothoracic ratio of 0.67 (normal value < 0.6) **(E)**. Echocardiography showed atrial septal defect, mitral regurgitation, tricuspid regurgitation and dilatation of the aortic root, 15 mm with Z-score of 4.48 **(F–H)**.

The Chinese female patient was the third-born child of a nonconsanguineous couple, and her siblings were unaffected. She was born at 39 weeks of gestational age by spontaneous vaginal delivery. She had normal birth measurements: her weight was 3.1 kg, her length was 50 cm and her head circumference was 34 cm. She was referred to the clinic at 2 months of age because of pneumonia and congestive heart failure. She displayed distinctive facial features including bilateral temporal skull flattening, enophthalmos and retrognathia (Figures 1A, B), and skeletal anomalies including arachnodactyly, pes planus, long, narrow feet, hammer toes, skin striae (Figures 1C, D), scoliosis and pectus excavatum. Positive wrist and thumb signs, reduced extension at elbows and joint hypermobility were observed. X-ray showed significant enlargement of the heart shadow with a cardiothoracic ratio of 0.67 (normal value < 0.6) (Figure 1E). Echocardiography showed atrial septal defect, mitral regurgitation, tricuspid regurgitation and dilatation of the aortic root, 15 mm with Z-score of 4.48 (Figures 1F–H). The combination of aortic root dilatation and 10 points of systemic features resulted in the clinical diagnosis of MFS, based on a set of manifestations from the revised Ghent nosology (Loeys et al., 2010).

### Genetic analysis

**FIGURE 2:**
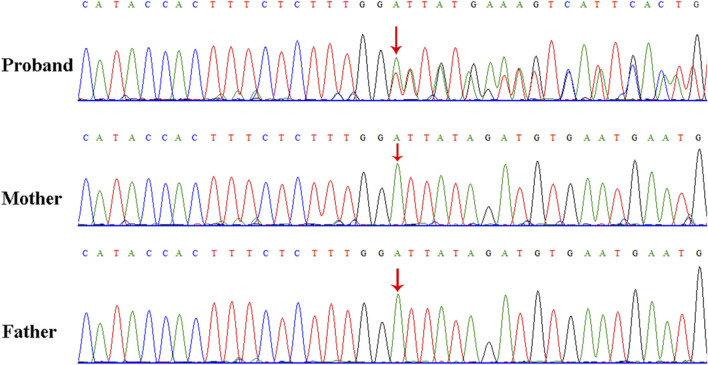
Sanger sequencing results for the patient, and the patient’s father and mother. The analysis demonstrated the presence of a deep intronic variant in *FBN1* (c.4943-8_4943-7insTATGTGATATTCATTCAC) in the patient and the absence of the variant in her parents. The red arrow indicates the variant site.

**FIGURE 3:**
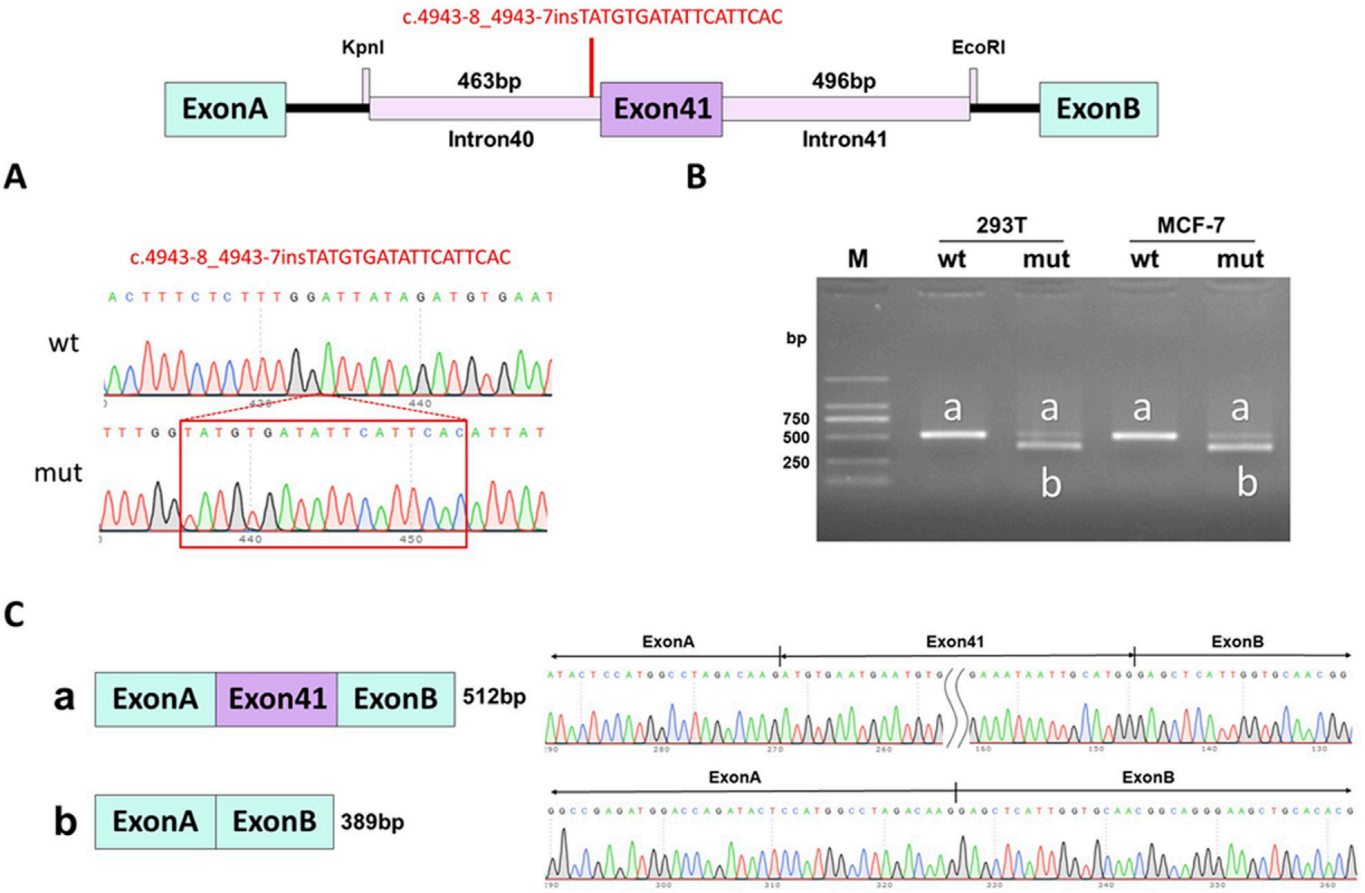
Minigene assay for *FBN1* c.4943-8_4943-7insTATGTGATATTCATTCAC variant and schematic diagram of the splicing pattern. **(A)** The construction of FBN1-pcMINI minigene plasmid; **(B)** Gel electrophoresis of RT-PCR revealed a single band for wild-type (wt) and two bands for mutant-type (mut); **(C)** minigene product sequencing demonstrated that the wild-type minigene formed normal mRNA, but the c.4943-8_4943-7insTATGTGATATTCATTCAC variant in *FBN1* caused a splicing abnormality, which abrogates the canonical splice site of intron 40, resulting in exon 41 skipping.

WES identified a novel deep intronic variant in intron 40 of *FBN1*, (NM_000138.4:c.4943-8_ 4943-7insTATGTGATATTCATTCAC) in the patient. Sanger sequencing confirmed the variant and the variant was absent from the parents, thus it was a *de novo* event (Figure 2) (PS2). In addition, the variant was not present in the Genome Aggregation Database, the 1000 Genomes Project or our internal database (PM2_supporting). Four computational splicing tools (Human Splicing Finder, SpliceAI, ESE Finder v3.0, NetGene2) predicted that this deep intronic variant could lead to use of a new splice acceptor site within intron 40 of the *FBN1* transcript. To further assess the impact of this variant, minigene study was performed to investigate the transcriptional outcome of the variant identified. An FBN1-pcMINI minigene was constructed and the cDNA of the wild-type and mutant mRNAs was obtained via RT-PCR. We observed that the variant affected splicing and caused exon 41 skipping*.* The final annotation was an infame deletion variant: c.4943_5065del, p.Val1649_Asp1689del. (PM4_strong) (Figure 3). Moreover, the patient’ manifestations were highly consistent with that of MFS (PP4). Furthermore, WES did not identify any other variants in *FBN1* and also excluded other possible known genetic causes. Thus, this variant was evaluated as clinical pathogenic according to the ACMG/AMP guidelines (PS2 + PM2_supporting + PM4_strong + PP4) (PS: pathogenic strong; PM: pathogenic moderate; PP: pathogenic supporting).

## Discussion

The *FBN1* gene is located on 15q21.1, consists of 66 exons and encodes the large molecule fibrillin-1 of 2,871 amino acids. Fibronectin-1 is widespread in connective tissues, and the protein contains 47 cysteine-rich epidermal growth factor EGF-like repeats and seven transforming growth factor-β1 binding protein-like domains (Ramirez and Dietz, 2007). *FBN1* mutations cause MFS through haploinsufficiency or dominant-negative effects mechanism (Aoyama et al., 1994; Whiteman et al., 2001; Faivre et al., 2007; Mátyás et al., 2007). Interestingly, studies have shown that missense mutations in exon 41 or 42 of *FBN1* cause geleophysic dysplasia (GD, MIM #614185) or acromicric dysplasia (AD, MIM #102370) through a gain-of-function mechanism, which is characterized by severe short stature, short hands and feet, joint stiffness, and skin thickening, but without cardiac involvement or early death (Le Goff et al., 2011; Passarge et al., 2016).

Many different types of *FBN1* variants have been identified. *FBN1* null variants (frameshift, nonsense and canonical variants) and missense variants are frequently detected in MFS patients (Robinson et al., 2006). However, deep intronic variants are rarely reported in MFS patients because these variants were generally considered to have no deleterious effect on protein product, thus they were easily to be missed during variant interpretation. Here, we identified a novel *de novo* deep intronic variant in *FBN1* (c.4943-8_4943-7insTATGTGATATTCATTCAC) in a 2-month-old female patient with clinical features suggestive of MFS. This variant was initially considered as a variant of unknown significance according to ACMG/AMP guideline. Since the patient met the clinical diagnostic criteria for MFS, the deep intronic variant was considered as a candidate variant. Multiple computational splicing tools predicted that the variant could cause use of a new splice acceptor site within intron 40 of the *FBN1* transcript. In order to seek the convincing evidence, *in vitro* minigene testing was performed and showed that the variant abrogates the canonical splice site of intron 40, resulting in exon 41 skipping. Eventually, the variant was annotated as an inframe deletion variant (c.4943_5065del, p.Val1649_Asp1689del), which was evaluated as clinical pathogenic according to the ACMG/AMP guidelines. Thus, the patient was clinically and molecularly diagnosed with MFS.

**FIGURE 4:**
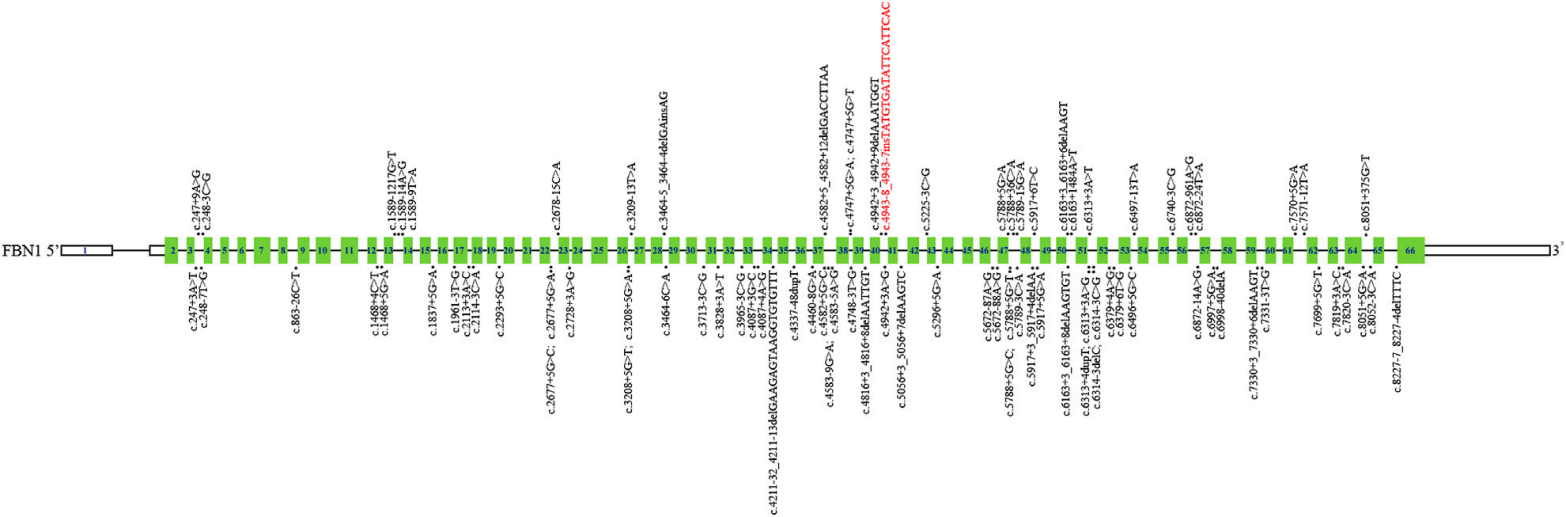
Schematic representation of *FBN1* deep intronic variants identified to date. The structure of *FBN1* contained 66 exons (blue rectangles), introns (black horizontal line); The localization of variants identified is depicted with dots. The dots above *FBN1* indicate deep intronic variants determined by functional analysis, and those below *FBN1* indicate deep intronic variants not determined by functional analysis. Red: Novel variants identified in this study.

Then, we systematically reviewed and analyzed deep intronic variants in *FBN1* (Biggin et al., 2004; Gillis et al., 2014; Xiong et al., 2015; Groth et al., 2017; Fusco et al., 2019; Wai et al., 2020; Guo et al., 2023; Bai et al., 2024); [HGMD database]. Currently, a total of 85 deep intronic variants, including the novel variant identified in our study, were identified (Figure 4). Among these variants, 28 of 86 (32.6%) have been verified to be pathogenic through functional analysis such as patient’s mRNA expression or *in vitro* minigene assays. Thus, additional investigations are needed to determine the pathogenicity of the other deep intronic variants. No obvious mutation spots were observed. Certainly, it is also necessary to collect more cases with deep intronic variants in *FBN1* to enrich mutation spectrum of *FBN1*.

In conclusion, we identified a novel deep intronic variant in *FBN1* in a Chinese patient diagnosed with MFS. Our findings expanded *FBN1* mutation spectrum, and highlighted that deep intronic variants should not be neglected in the interpretation of variants, and may be a potential cause of disease. Additional functional studies are necessary to verify the pathogenicity of deep intronic variants.

## Data Availability

The data presented in the study are deposited in NODE (https://www.biosino.org/node) with the accession number OEP00006104 or through the URL: https://www.biosino.org/node/project/detail/OEP00006104.

## Abbreviations

MFS, Marfan syndrome; HEK 293T, human embryonic kidney 293T; GD, geleophysic dysplasia; AD, acromicric dysplasia; WES, Whole-exome sequencing.
